# Toll-like receptor agonist therapy can profoundly augment the antitumor activity of adoptively transferred CD8^+^ T cells without host preconditioning

**DOI:** 10.1186/s40425-016-0110-8

**Published:** 2016-02-16

**Authors:** Michelle H. Nelson, Jacob S. Bowers, Stefanie R. Bailey, Marshall A. Diven, Caroline W. Fugle, Andrew D. M. Kaiser, Claudia Wrzesinski, Bei Liu, Nicholas P. Restifo, Chrystal M. Paulos

**Affiliations:** Microbiology and Immunology, Hollings Cancer Center, Medical University of South Carolina, Charleston, SC 29425 USA; Center for Cancer Research, National Cancer Institute (NCI), National Institutes of Health (NIH), Bethesda, MD 20892 USA

**Keywords:** Total body Irradiation, Adoptive immunotherapy, CD8^+^ T lymphocytes, Innate immunity

## Abstract

**Background:**

Lymphodepletion enhances adoptive T cell transfer (ACT) therapy by activating the innate immune system via microbes released from the radiation-injured gut. Microbial components, such as LPS, are key mediators of total body irradiation (TBI) enhancement, but our ability to strategically use these toll-like receptor (TLR) agonists to bolster the potency of T cell-based therapies for cancer remains elusive. Herein, we used TLR4 agonist LPS as a tool to address how and when to use TLR agonists to effectively improve cancer immunotherapy.

**Methods:**

To determine the mechanisms of how innate immune activation via lymphodepletion potentiated antitumor T cell immunity, we utilized the pmel-1 melanoma mouse model. B16F10-bearing mice were preconditioned with 5Gy TBI and given a tripartite ACT therapy (consisting of transferred pmel-1 CD8^+^ T cells, vaccination with fowlpox encoding gp100, and IL-2) along with TLR4 agonist LPS. The timing of LPS administration and the requirement of individual components of the tripartite therapy were evaluated based on tumor growth and the phenotype of recovered splenocytes by flow cytometry. We also evaluated the role of non-toxic and clinically used TLR4 and TLR9 agonists—monophosphoryl lipid A (MPL) and CpG Oligodeoxynucleotide (CpG ODN), respectively— for ACT therapy.

**Results:**

Here we report that while exogenous administration of LPS was able to enhance adoptively transferred CD8^+^ T cells’ tumor destruction, LPS treatment alone did not replace individual components of the tripartite ACT regimen, or obviate TBI. Moreover, we found that sequentially administering LPS during or one day prior to ACT therapy compromised tumor regression. In contrast, administering LPS after ACT potentiated the antitumor effectiveness of the regimen, thereby supporting the expansion of transferred tumor-specific CD8^+^ T cells over host CD4^+^ T cells. We also found that non-toxic TLR agonists MPL and CpG potentiated the antitumor activity of infused CD8^+^ T cells. Finally, TBI was no longer needed to regress tumors in mice who were depleted of host CD4^+^ T cells, given a tripartite ACT regimen and then treated with low dose LPS.

**Conclusions:**

Collectively, our results identify how and when to administer TLR agonists to augment T cell-based immunotherapy in the absence or presence of host preconditioning for treatment of advanced malignancies. Our findings have clinical implications for the design of next generation immune-based therapies for patients with cancer.

**Electronic supplementary material:**

The online version of this article (doi:10.1186/s40425-016-0110-8) contains supplementary material, which is available to authorized users.

## Background

The human digestive tract contains 100 times more single-celled organisms than the sum total of all cells within the body [1]. The microbiome has evolved in symbiotic ways to play a key role in sustaining human health, including the absorption of nutrients, maintenance of mucosal integrity and the regulation of intestinal homeostasis [2–5]. Homeostatic disruption can induce microbial translocation, triggering a switch in the commensal host-microbe relationship from mutualistic to pathogenic [6, 7]. This phenomenon exacerbates graft-versus-host disease, inflammatory bowel disease and HIV/AIDS infection [8–10]. Surprisingly, the deleterious effect of this phenomenon via chemo- and/or radiotherapy is beneficial for T cell-based treatments for cancer; including adoptive T cell transfer (ACT) therapy [11–14].

Lymphodepletion with chemotherapy and/or radiation preparative regimens administered prior to adoptive immunotherapy mediates objective clinical responses in ~50 % of patients with metastatic melanoma and has recently shown promise in metastatic cervical cancer [15–17]. Likewise, ACT treatment with anti-CD19 chimeric antigen receptor (CAR)-engineered T cells mediates robust tumor immunity in preconditioned patients with hematological malignancies [18–22]. Additionally, tumor mutation-specific T cells are now being exploited for ACT therapy [23]. How lymphodepletion augments ACT in these various clinical trials has been elucidated in clinically relevant mouse models of melanoma.

A non-myeloablative lymphodepleting preparative regimen with 5Gy TBI prior to an ACT regimen can induce destruction of B16F10 melanoma in mice by removing cytokine sinks, depleting suppressive T_reg_ cells, transiently ablating myeloid derived suppressor cells (MDSCs) and activating the innate immune system [11]. Interestingly, innate immune activation via microbial LPS and other microbial agonists, liberated from the radiation-injured gut, was responsible for triggering toll-like receptor 4 (TLR4) signaling and is a mechanism underlying the enhanced ACT effectiveness in mice [11, 24–27]. Removal of signaling components, through the use of mice deficient in TLR4, reduced the beneficial effect of TBI [11]. Likewise, cancer patients who carry a TLR4 loss-of-function allele relapsed more quickly after chemotherapy than those carrying the normal TLR4 allele [28]. Additional investigation revealed that increasing the intensity of irradiation from 5 to 9Gy TBI, which requires hematopoietic stem cell (HSC) support, further enhanced treatment outcome [29]. Collectively, these findings illuminate an important role for innate immunity in augmenting T cell-based tumor immunity.

Microbes released from the radiation-injured gut contain a plethora of TLR ligands besides LPS. Any of these ligands may contribute to activating the innate immune system. However, herein, we used TLR4 agonist LPS as a tool to address how and when to use adjuvants to improve cancer immunotherapy. We asked if TLR4 agonist LPS could replace and/or enhance vaccines, transferred CD8^+^ T cells and/or host lymphodepletion in an aggressive model of melanoma. We found that activating the innate immune system in non-irradiated mice with exogenously delivered LPS could not replicate the effectiveness of TBI when used in conjunction with ACT therapy. Yet, exogenous LPS could augment the antitumor activity of infused CD8^+^ T cells in mice receiving a non-myeloablative, low level of irradiation (5Gy TBI). We also found that administering LPS after, but not before or simultaneous with, ACT treatment was optimal for potentiating the antitumor activity of infused CD8^+^ T cells. Additional investigation revealed that co-administration of LPS plus antibody depletion of host CD4^+^ T cells triggered robust tumor eradication. Surprisingly, this combination eliminated the previous requirement for TBI. LPS is not the only TLR agonist capable of enhancing ACT. We found that the use of MPL or CpG in similar conditions also augmented ACT. Collectively, these findings provide insight into how to use other TLR agonists with ACT regimens and suggests alternative reagents to lymphodepletion that might safely treat patients sensitive to chemotherapy/radiotherapy.

## Results

### Lymphodepletion augments transferred antitumor CD8^+^ T cells by activating DCs and ablating host lymphocytes

**Fig. 1:**
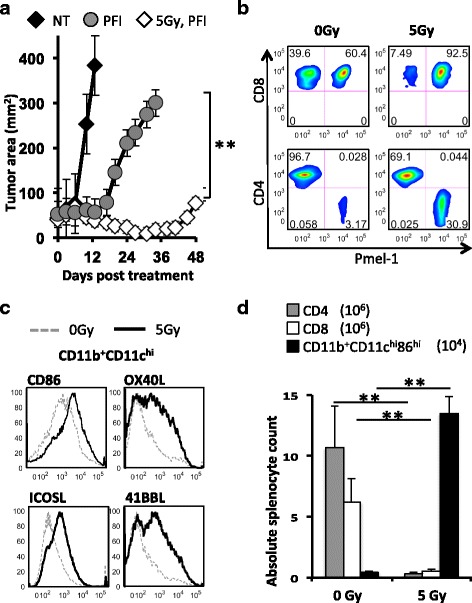
Lymphodepletion augments the antitumor activity of transferred CD8^+^ T cells via depleting host suppressor T cells and activating APCs. **a** TBI augmented antitumor responses in mice. C57BL6 mice bearing subcutaneous B16F10 tumors established for 8 days received non-myeloablative 5Gy TBI or were not irradiated. One day later, mice received an ACT treatment regimen consisting of the adoptive transfer of 1e^6^ cultured tumor-reactive pmel-1 CD8^+^ T cells, fowlpox hgp100 vaccination and hIL-2 or were left untreated (NT). Data (mean +/- SEM, *n* = 5 mice per group) are representative of 5 independent experiments. PFI vs. 5Gy PFI, **P < .01, ANOVA. **b** TBI increased the engraftment of infused pmel-1 CD8^+^ T cells over host CD8^+^ or host CD4^+^ T cells relative to non-irradiated mice. Ratios of transferred pmel-1 CD8^+^ T cells (Vβ13^+^Ly5.1^+^) relative to returning host CD8^+^ and CD4^+^ T cells are shifted toward pmel-1 CD8^+^ T cells in irradiated mice. B6 mice were given 5Gy TBI or not followed by the transfer of 1e^6^ pmel-1 CD8^+^ T cells (Ly5.1^+^), fowlpox hgp100 vaccination and hIL-2. Splenocytes obtained 1 week after transfer were simultaneously analyzed for infused pmel-1 CD8^+^ and reconstituting host cells. **c** TBI up-regulates costimulatory molecules on host CD11b^+^CD11c^hi^ dendritic cells. Splenic DCs were isolated at 24 h from mice treated with 5Gy TBI or without. The expression of costimulatory molecules (CD86, ICOSL, OX40L and 41BBL) on CD11c^hi^-gated DCs were analyzed by flow cytometry and displayed in histogram form. **d** TBI depletes endogenous CD4^+^ and CD8^+^ T cells and transiently promotes activation of CD11c^hi^ dendritic cells. Splenocytes were isolated from 0 and 5Gy irradiated mice 2 days after TBI. Absolute numbers of CD4^+^, CD8^+^ and activated CD11b^+^CD11c^hi^CD86^hi^ DCs in the spleens of TBI and non-irradiated C57BL6 mice were enumerated. Data shown are representative of 2 independent experiments. **P < .01, unpaired *t*-test

Lymphodepletion with 5Gy TBI enhances a tripartite ACT treatment consisting of PFI (P = infusion of 1e^6^ pmel-1 CD8^+^ T cells, F = vaccination with fowlpox encoding hgp100 and I = high dose IL-2) in mice with B16F10 melanoma to a greater extent than in lymphoreplete mice (Fig. 1a). We evaluated how lymphodepletion impacts the activation of the innate immune system and the degree of pmel-1 CD8^+^ T cell engraftment vs. host cell depletion. The improved outcomes in 5Gy TBI + PFI treated mice compared to non-irradiated + PFI treated mice correlated with a higher relative frequency of donor pmel-1 CD8^+^ T cells in the tumor (Fig. 1b). Moreover, we found that irradiated mice had an increased number of activated DCs expressing the co-stimulatory molecules CD86, OX40L, ICOSL and 41BBL; likely promoting the proliferation of infused CD8^+^ lymphocytes (Fig. 1c). Given that 5Gy TBI decreases the cellular density in mice over time, we evaluated the absolute number of immune cells following TBI. We found that increasing the intensity of lymphodepletion from 0 to 5Gy TBI was associated with a greater reduction in the absolute number of splenic host CD4^+^ and CD8^+^ T cells (Fig. 1d; Day 3 CD4^+^ and CD8^+^ cells: 0Gy TBI-10.6 and 6.45e^6^; 5Gy TBI-0.35 and 0.56e^6^, respectively). Consistent with previous work [11, 30], the absolute number of activated dendritic cells in the spleen transiently increased as the intensity of irradiation was increased from 0 to 5Gy TBI (Fig. 1d; Day 1 CD11b^+^CD11c^hi^CD86^hi^ cells: 0Gy TBI-0.5e^4^; 5Gy TBI-13.5e^4^). Collectively, these data revealed that the addition of TBI correlated with a greater depletion of endogenous lymphocytes and activation of the innate immune system.

### The antitumor activity of transferred CD8^+^ T cells is compromised in MyD88 deficient mice post-irradiation

**Fig. 2:**
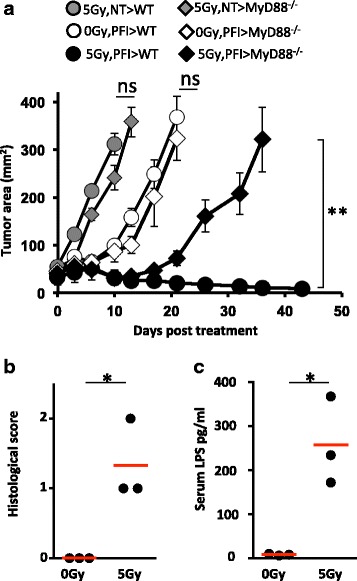
TLR-MyD88 signaling triggered by TBI is critical for augmenting the antitumor activity of transferred CD8^+^ T cells. Tumor bearing mice were given tripartite ACT therapy consisting of an infusion of 1e^6^ transgenic pmel-1 CD8^+^ T cells with a TCR that recognizes the gp100 peptide on B16 tumors, a viral vaccination encoding gp100 peptide and IL-2 cytokine support in WT versus MyD88 deficient animals with or without 5Gy TBI. **a** The effectiveness of treatment was decreased in irradiated mice genetically deficient in MyD88. WT and MyD88^–/–^ tumor-bearing mice were irradiated and then received ACT treatment or were left untreated. Data (mean ± SEM; 5 mice per group) are representative of 2 independent experiments. 5Gy PFI > WT vs. 5Gy PFI > MyD88^-/-^, **P < .01, ANOVA. **b** TBI compromises the colon. Colon of mice were analyzed at 3 days post-TBI and scored by a pathologist unaware of the treatment groups. Data shown (3 mice per group) are representative of 5 independent experiments. *P < .05, unpaired t test. **c** TBI promotes translocation of gut-derived LPS. Serum from non-irradiated and 5Gy irradiated mice were collected and analyzed for the presence of LPS using a LAL assay 6 days after TBI. Data shown (3 mice per group) are representative of 5 independent experiments. *P < .05, unpaired *t*-test

We reported that TLR4 signaling was critical for a robust antitumor response, as TLR4 knockout mice had reduced antitumor benefits following TBI [11]. As other innate microbes and changes in gut microbes in general, likely triggered by TBI, were responsible for activating the innate immune system, we sought to elucidate if global ablation of MyD88 (a universal adapter protein used by almost all TLRs (except TLR3) to activate transcription factor NF-κB [31]) signaling would also impair treatment outcome by ACT therapy in lymphodepleted animals. To address this question, we examined the consequences of a tripartite ACT therapy (PFI) in WT versus MyD88 deficient animals with or without 5Gy TBI. Tumor destruction was significantly impaired in irradiated MyD88^-/-^ mice (*P* < 0.02; black diamond vs. black circle) compared to irradiated WT mice (Fig. 2a). Conversely, we observed no difference between the effectiveness of ACT tumor treatment in non-irradiated WT or MyD88^-/-^ mice (Fig. 2a). Additionally, we discovered that TBI damages the integrity of the gut by pathological score (Fig. 2b) and permitted microbial translational in irradiated mice, as detected by LPS in the serum of irradiated mice 6 days after TBI (Fig. 2c). Our preliminary work shows that LPS can be detected confidently at days 5 to 7, but not before this time point (not shown). Thus, our data identified an essential role for MyD88 in the induction and stability of antitumor CD8^+^ T cells in irradiated mice. We surmised that microbial TLR agonist (such as LPS and beyond) enhanced ACT tumor treatment via MyD88-dependent signaling in irradiated animals only.

### Administration of LPS after ACT enhances antitumor immunity only in irradiated animals

**Fig. 3:**
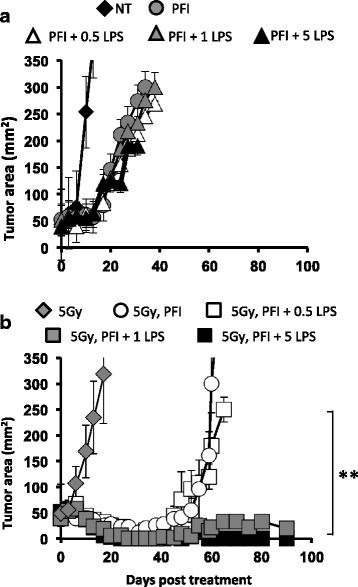
Administration of LPS enhances antitumor immunity in irradiated but not lymphoreplete mice. **a** LPS does not augment antitumor responses in non-irradiated mice. Mice bearing subcutaneous B16F10 tumors were established for 8 days. Mice received an ACT treatment comprised of the adoptive transfer of 5e^5^ cultured pmel-1 T cells, fowlpox hgp100 vaccination and hIL-2 or were left untreated. The next day, mice received ultra-pure LPS ranging from 0.5 to 5 μg or left untreated. Data shown (mean ± SEM, 10 mice per group) are representative of 2 independent experiments. PFI vs. PFI + 0.5, 1 or 5 LPS, NS, ANOVA. **b** LPS augments the antitumor activity of pmel-1 CD8^+^ T cells in irradiated mice. Mice bearing subcutaneous B16F10 tumors established for 8 days received 5Gy TBI. One day after TBI, mice received an ACT treatment comprised of the adoptive transfer of 5e^5^ cultured pmel-1 T cells, fowlpox hgp100 vaccination and hIL-2 or were left untreated. The next day, mice received LPS ranging from 0.5 to 5 μg or left untreated. Data shown (mean ± SEM, 5–10 mice per group) are representative of 2 independent experiments. 5 Gy PFI (white circle) vs. 5Gy PFI + 0.5 LPS (white square), NS. ANOVA. 5Gy PFI + 0.5 LPS (white square) vs. 5 Gy PFI + 1 or 5 LPS (grey or black square), ***P < .001, ANOVA

Because microbial LPS was detected in the sera of irradiated animals (Fig. 2c), we used TLR4 agonist LPS as a tool to address how and when to use TLR agonists to potentially improve adoptive T cell transfer cancer immunotherapy. We posited that administering ultrapure LPS to non-irradiated mice would bypass the previous need for TBI to augment ACT therapy. We first determined the highest dose of LPS that could be tolerated in non-irradiated mice given ACT therapy. To this end, increasing doses of ultrapure LPS, ranging from 0.1 to 10 μg, were administered to non-irradiated animals one day after an ACT and PFI therapy and their tolerance was monitored by their overall appearance and survival. In contrast to our hypothesis, we found that even the highest tolerable dose of LPS (5 μg) administered to non-irradiated mice could not enhance the treatment or their survival (Fig. 3a). Treating mice with 10 μg of LPS was toxic to the mice with limited survival 2 days after its administration (not shown). Collectively, these data showed that merely increasing the dose of LPS to the highest tolerable dose was not sufficient to improve ACT in non-irradiated animals compared with ACT in irradiated mice.

We next sought to determine what dose of LPS might safely and most effectively enhance ACT treatment in animals given 5Gy TBI. Thus, LPS doses ranging from 0.1 to 25 μg were administered to irradiated animals one day after treatment. In contrast to our findings in non-irradiated animals, 1 μg of LPS could significantly potentiate CD8^+^ T cell-mediated tumor eradication in irradiated animals. Likewise, doses of LPS exceeding 1 μg of LPS improved ACT treatment (Fig. 3b) and doses lower than 1 μg of LPS did not augment ACT treatment. Interestingly, LPS doses that were toxic in non-irradiated mice were well tolerated in irradiated hosts given ACT treatment. Thus, 25 μg of LPS, a dose highly toxic to all non-irradiated mice, was well tolerated in irradiated animals (not shown). The reduced toxicity observed in irradiated, but not in non-irradiated, mice given LPS might be due to the fact that TBI ultimately reduces (transiently) the absolute number of innate immune cells triggered by LPS [32]. Collectively, our data revealed that LPS greatly improves treatment outcome, but only in conjunction with TBI.

### LPS induces CD25 on antitumor CD8^+^ T cells and supports their persistence

**Fig. 4:**
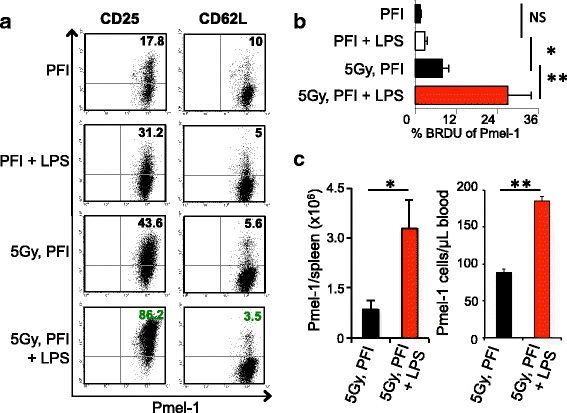
LPS enhances CD25 expression on transferred CD8^+^ T cells and improves their long-term persistence in irradiated animals. **a** LPS enhances the expression of CD25, but not CD62L, on adoptively transferred pmel-1 CD8^+^ T cells (Ly5.1^+^) when analyzed on day 7 from irradiated B6 mice given fowlpox hgp100 and hIL-2 as demonstrated by flow cytometry. **b** LPS enhances the initial proliferation of adoptively transferred cells as indicated via BrdU incorporation. On day 3 post-ACT, mice were given 1 mg of BrdU and sacrificed 2 h later. Transferred cells were analyzed for BrdU uptake (5 mice per group). **c** LPS increased the absolute number of transferred pmel-1 T cells in the spleen and blood of irradiated hosts. Absolute numbers of transferred pmel-1 cells (CD8^+^Ly5.1^+^) in the spleens and blood were enumerated from Thy1.1 mice. Data shown (mean ± SEM, 5 mice per group) are representative of 2 independent experiments. *P < .05, **P < .01, unpaired *t*-test

How exogenous LPS impacts the phenotypic signature and proliferative capacity of infused pmel-1 CD8^+^ T cells in mice given TBI remains incompletely elucidated. Thus, we investigated how LPS influenced the expression of CD62L, CD44 and CD25 on transferred T cells in non-irradiated and irradiated mice 5 days post-infusion. Interestingly, LPS doubles the expression of CD25, a receptor for IL-2, on all transferred cells, regardless of therapy, but irradiation clearly enhances CD25 expression (86 %) on infused donor pmel-1 T cells (non-irradiated, 31 %; Fig. 4a). These data imply that infused tumor-specific CD8^+^ T cells from irradiated mice given LPS might have an advantage in consuming the homeostatic cytokine IL-2 in vivo. In contrast, there were no differences in the expression of CD62L (Fig. 4a) and CD44 (not shown) on the transferred T cells in irradiated or non-irradiated mice given LPS. To investigate how LPS impacts the in vivo proliferative capacity of the infused pmel-1 CD8^+^ T cells, we treated the mice with BrdU and determined its percent incorporation into infused pmel-1 cells 3 days post-transfer. We found that the transferred cells from irradiated mice given LPS incorporated significantly more BrdU than in mice receiving TBI alone (Fig. 4b). Moreover, LPS was less effective at driving the proliferation of infused CD8^+^ T cells in non-irradiated animals. These data might imply that ablating suppressive lymphocytes with TBI while concomitantly intensifying innate activation through administration of a higher concentration of LPS to the host unmasked the proliferative capacity of the transferred pmel-1 CD8^+^ T cells. However, additional experiments will be needed to confirm this postulation. Also, it will be insightful to know whether the expression of receptors for IL-7, IL-15 and IL-21 on donor T cells were up-regulated following TBI. The absolute number of pmel-1 CD8^+^ T cells was also considerably elevated in the spleen and blood of irradiated mice receiving LPS (compared with irradiated mice not receiving LPS) 35 days after treatment (Fig. 4c). Collectively, our data indicate that administering LPS to irradiated animals drives the proliferative capacity and increases the persistence of transferred cells.

### Infused CD8^+^ T cells, vaccine and IL-2 are required to eradicate melanoma in irradiated mice treated with LPS

**Fig. 5:**
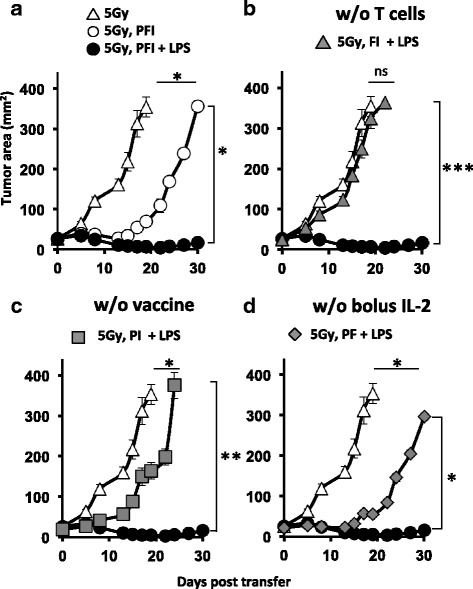
Vaccination, bolus IL-2 and infusion of tumor-reactive lymphocytes are required to potentate ACT tumor treatment in irradiated mice given LPS. **a** LPS augmented the antitumor activity of CD8^+^ T cells in irradiated mice given the tripartite treatment. **b** Removal of pmel-1 T cells; **c** fowlpox hgp100 vaccine or **d** bolus IL-2 impaired the enhanced antitumor response mediated in irradiated mice given a tripartite therapy and LPS. One day after TBI, mice received an ACT treatment comprised of the adoptive transfer of 5e^5^ cultured pmel-1 T cells, fowlpox hgp100 vaccination and hIL-2 or were left untreated. The next day, mice received 2 μg of LPS or left untreated. Data shown (mean ± SEM, 5–10 mice per group) are representative of 2 independent experiments. 5Gy (white triangle) vs. 5Gy PFI (white circle, *P < .05), 5Gy FI+ LPS (grey triangle, NS), 5Gy PI + LPS (grey square, *P < .05) or 5Gy PF + LPS (grey diamond, *P < .05), ANOVA. 5Gy PFI + LPS (black circle) vs. 5Gy PFI (white circle, *P < .05), 5Gy FI + LPS (grey triangle, ***P < .001), 5Gy PI + LPS (grey square, **P < .01) or 5Gy PF + LPS (grey diamond, *P < .05), ANOVA

Because LPS bolstered the persistence of infused CD8^+^ T cells and mediated curative responses in irradiated mice, we hypothesized that LPS administration might drive tumor regression without the need for vaccination or IL-2. To address this idea, we replaced infused tumor-specific CD8^+^ T cells, vaccination via fowlpox expressing hpg100 or high dose IL-2 with LPS in irradiated mice. Although administration of LPS reproducibly mediated tumor regression in irradiated mice receiving the tripartite ACT treatment, LPS did not replace individual components of the regimen (Fig. 5). Indeed, replacing infused cells or vaccination with LPS in the tripartite regimen impaired the treatment achieved in irradiated mice given the tripartite regimen (Fig. 5b and c, respectively). In contrast, replacing IL-2 with LPS was comparable to treatment seen in irradiated mice only given the tripartite regimen (in other words: 5Gy PFI (Fig. 5a; white circle) = 5Gy PF + LPS (Fig. 5d; grey diamond)), implying that LPS might improve the capacity of transferred cells to acquire endogenous IL-2. This idea is plausible given our finding that LPS increased CD25 expression on infused CD8^+^ T cells (Fig. 4a). Nonetheless, replacing IL-2 with LPS was less effective than treatment driven by LPS in irradiated mice receiving the entire tripartite regimen (5Gy, PFI + LPS; black circle)-which was the most efficacious treatment. Collectively, we found that LPS administration enhanced ACT therapy in irradiated mice but did not replace individual components of the regimen.

### LPS administration, relative to ACT therapy, impacts treatment outcome

TLR agonists are used clinically alone or in combination with tumor vaccines [33–37]. Although TLR agonists plus vaccine combinations have shown success in triggering T cell immune responses in patients, they have not consistently mediated tumor regression [38]. Interestingly, when testing TLR agonist combinations with different therapeutic strategies, investigators have not determined the optimal timing for administering TLR agonists relative to vaccines, checkpoint modulators and/or T cell-based therapies [39].

**Fig. 6:**
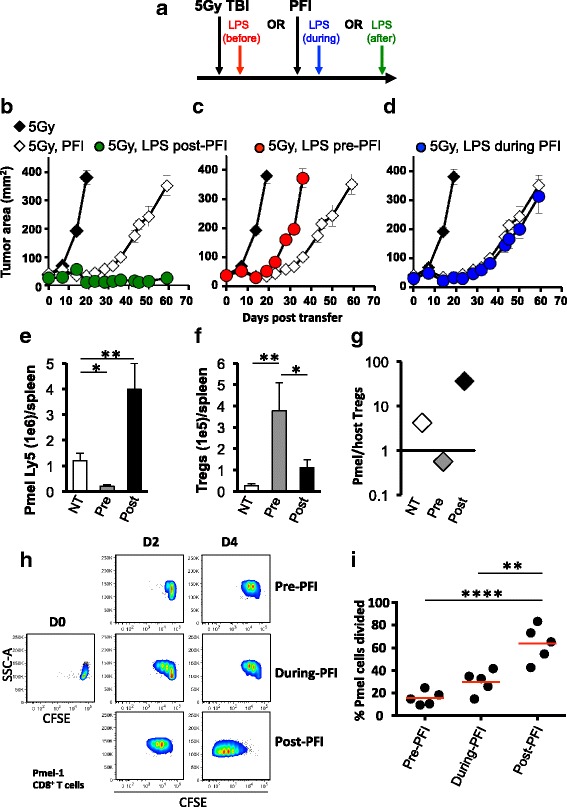
The timing by which LPS is administered relative to ACT therapy differentially impacts treatment outcome and regulates the innate and adaptive immune system. **a** Schematic showing the time at which LPS is administered to tumor bearing mice relative to the tripartite ACT treatment regimen. One day after TBI, mice received an ACT treatment comprised of the adoptive transfer of 5e^5^ cultured pmel-1 T cells, fowlpox hgp100 vaccination and hIL-2 or were left untreated. Either on day prior (**b**), during (**c**) or one day after (**d**) ACT, mice received 2 μg of LPS or were left untreated. Data shown (mean ± SEM, 5 mice/group) are representative of 4 independent experiments. 5Gy (black diamond) vs. 5Gy PFI (white diamond, **P < .01), 5Gy PFI pre-LPS (red circle, *P < .05), 5Gy LPS during PFI (blue circle, **P < .01) or 5Gy PFI post-LPS (green circle, **P < .01), ANOVA. 5Gy PFI (white diamond) vs. 5Gy PFI pre-LPS (red circle, *P < .05), 5Gy LPS during PFI (blue circle, NS) or 5Gy PFI post-LPS (green circle, **P < .01), ANOVA. (**e**–**g**) Splenocytes were harvested from irradiated mice on day 7 post-T cell infusion and absolute cell counts were determined by flow cytometry. *P < .05, **P < .01, unpaired *t*-test. (**h & i**) CD11c^hi^ dendritic cells were sorted by flow cytometry from single-cell B16F10 tumor suspensions prepared 6 days after ACT treatment from mice given LPS at different time points (as shown in scheme A). Tumor-infiltrating DCs were co-cultured for 4 days with CFSE-labeled, negatively-isolated pmel CD8^+^ T cells at a 10:1 T cells: APC ratio. DCs were exposed to antigen during in vivo fowlpox vaccination. Tumor antigen was not added to the co-culture. Representative flow cytometry plots (**h**) and dot plots (**i**) of CFSE dilution of pmel-1 cells. ****P < .0001, **P < .01, ANOVA

Although we found that administering LPS after ACT therapy potentiates the effectiveness of the tripartite therapy in animals (for example Fig. 3b), it remained unknown whether this time-point was ideal to deliver TLR agonists relative to T cell infusion, vaccination and IL-2. To address this question, as depicted in Fig. 6a, we administered LPS either one day prior, during or one day after the tripartite ACT regimen and monitored tumor growth in irradiated animals. Consistent with previous experiments, administering LPS after ACT potentiated CD8^+^ T cell-mediated tumor regression (Fig. 6b). In stark contrast, treating mice with LPS one day before ACT treatment statistically impaired tumor growth and mice rapidly died (Fig. 6c). Administering LPS at the same time as ACT did not impact treatment outcome compared to ACT treatment alone (Fig. 6d).

We next sought to explore how the timing of LPS administration relative to the ACT regimen impacts the engraftment of infused pmel-1 CD8^+^ T cells versus host CD4^+^ T cells in vivo. We found that delivering LPS after the ACT regimen increased the absolute number of donor CD8^+^ T cells (Fig. 6e) to host CD4^+^ T cells (Fig. 6f) in the spleen of irradiated mice 7 days after the treatment regimen, which likely explains why this approach effectively regresses B16F10 tumor. Conversely, giving LPS before the regimen supported host CD4^+^ T cells over infused pmel-1 CD8^+^ T cells (Fig. 6e and f), which likely impaired the antitumor activity of CD8^+^ T cells. If LPS was given to mice after ACT, it increased the ratio of infused CD8^+^ T cells to host CD4^+^ T cells in the tumor of mice (Fig. 6g). In contrast, a high ratio of host CD4^+^ T cells to infused CD8^+^ T cells was observed in the tumor of mice given LPS prior to the ACT tripartite regimen. Note, our preliminary data show that there are more host CD25^high^FOXP3+ CD4^+^ T cells and less donor pmel-1 CD8^+^ T cells in irradiated mice given LPS before PFI versus in mice given LPS after PFI (Additional file 1 A and B). These data indicate that the timing in which TLR agonists are used relative to ACT can dramatically impact treatment outcome, likely by altering the ratio of tumor-specific CD8^+^ T  cells to host immune cells.

### Dendritic cells residing within tumors of mice treated with LPS after a tripartite ACT regimen stimulate pmel-1 CD8^+^ T cell proliferation

Because LPS impaired the engraftment and antitumor activity of infused pmel-1 CD8^+^ T cells, we assumed that LPS prematurely activated host DCs and thus compromised their ability to take up, process and present antigen delivered via vaccination. To test this idea, we sought to observe the ability of tumor-residing DCs (from treatment groups in Fig. 6b, c, d) to stimulate the division of pmel-1 CD8^+^ T cells. Single cell suspensions of established melanoma from mice treated with LPS either before, during or after ACT therapy was sorted by flow cytometry for dendritic cells. Sorted DCs were then co-cultured with CFSE-labeled, pmel-1 TCR transgenic splenocytes at a 10:1 ratio of T cells: DCs. DCs isolated from tumors of mice treated with LPS prior to or during ACT regimen failed to robustly induce pme l-1 CD8^+^ T cell division (Fig. 6h). In contrast, DCs isolated from B16 tumors of mice treated with LPS after the tripartite ACT regimen stimulated strong in vitro CD8^+^ T cell proliferation within 4 days of co-culture (Fig. 6h). These findings show that DCs from mice given LPS after ACT promoted *ex vivo* proliferation of pmel-1 CD8^+^ T cells were significant and reproducible (Fig. 6i). Collectively, our data suggest that LPS potentiates the ability of DCs to drive pmel-1 CD8^+^ T cell responses to tumors in vivo when administered one day after the tripartite regimen.

Next, we sought to test our hypothesis that LPS beneficially increases co-stimulatory molecules only if given after PFI. We found that giving LPS to mice after ACT only slightly increased the expression of co-stimulatory molecules CD80 and CD86 on conventional DCs as well as on monocytes from the spleens of mice (3 days post ACT). Moreover, a minor increase in these molecules was induced on APCs if LPS was given before ACT (Additional file 1 C and D). We did not see an increase in co-stimulatory molecules 41BBL, OX40L or ICOSL on conventional DCs or monocytes by administering LPS to irradiated mice (either before or after PFI). Perhaps we did not see an increase in these particular molecules because TBI itself induces them. As shown in Fig. 1c, TBI induces these molecules, but they are lower on the APCs from non-irradiated cohorts. Collectively, our data imply that LPS slightly enhances DC activation, which might contribute to improving ACT therapy.

### Administration of MPL or CpG enhances antitumor immunity in irradiated mice

**Fig. 7:**
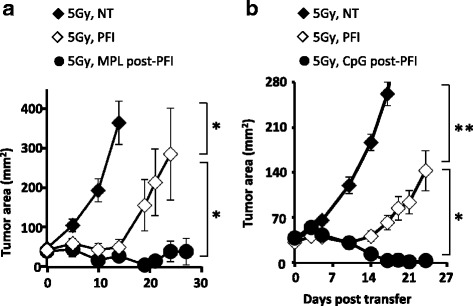
Administration of MPL or CpG enhances antitumor immunity in irradiated mice. Mice bearing subcutaneous B16F10 tumors established for 8 days received 5Gy TBI. One day after TBI, mice received an ACT treatment comprised of the adoptive transfer of 5e^5^ cultured pmel-1 T cells, fowlpox hgp100 vaccination and hIL-2 or were left untreated. The next day, mice received either (**a**) 5 μg MPL (i.v.) or (**b**) 10 μg of CpG (i.t.), daily for 4 days, or left untreated. Data shown (mean ± SEM, 5–10 mice per group) are representative of 2 independent experiments. For MPL treatment: 5Gy PFI vs. NT (*P < 0.05) or 5Gy MPL post-PFI (*P < 0.05), ANOVA. For CpG treatment: 5Gy PFI vs. NT (**P < 0.01) or 5Gy CpG post-PFI (*P < 0.05), ANOVA

Owing to its inherent toxicity, it is important to find an alternate agonist to LPS for tumor immunotherapy in the clinic. Moreover, some patients have TLR4 polymorphisms, rendering their innate immune system resistant to microbial LPS by chemotherapy or TBI [28]. Thus, we sought to determine whether TLR2/TLR4 monophospholipid A (MPL-a detoxified version of LPS) could also augment ACT treatment in irradiated hosts. Similar to ultrapure LPS, we found that MPL was effective in mediating tumor regression by the transferred cells (Fig. 7a). Importantly, we also found that another bacterial-derived agonist CpG-DNA (TLR9 agonist; Fig. 7b) augmented PFI treatment in irradiated mice. These data are important, as these agonists have been safely used in the clinic.

### Tumor eradication via ACT therapy can be achieved without host preconditioning

**Fig. 8:**
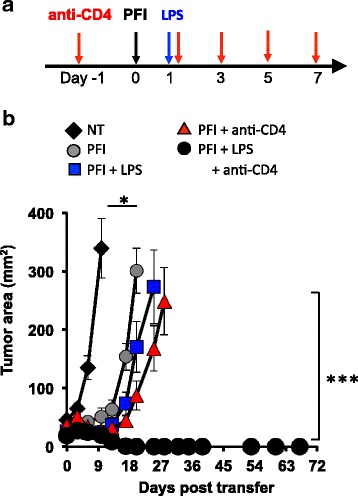
Long-term curative responses can be mediated in mice without lymphodepletion by depleting host CD4^+^ T cells and activating APCs with TLR4 agonists LPS. (**a**) Scheme for treating non-irradiated mice with CD4 depleting antibody, a tripartite ACT treatment and LPS. (**b**) Tumor eradication via ACT can be achieved without host preconditioning via antibody depleting CD4 lymphocytes that act as T_reg_ and cytokine sinks and activating innate immunity via TLR signaling. One day before ACT, mice were antibody depleted of host CD4^+^ T cells and subsequently administered every other day for a total of 5 doses. The ACT treatment regimen was comprised of the adoptive transfer of 5e^5^ cultured pmel-1 T cells, fowlpox hgp100 vaccination and hIL-2 or were left untreated. One day after ACT, mice received 2 μg of LPS or were left untreated. Data shown (mean ± SEM, 5 mice per group) are representative of 4 independent experiments. NT (black diamond) vs. PFI, PFI + LPS, or PFI + anti-CD4, *P < .05. PFI + LPS + anti-CD4 (black circle) vs. all groups *P* < 0.001, ANOVA

We next posited that lymphodepletion with 5Gy TBI or chemotherapeutics, which can have toxic side effects in patients, could be bypassed in animals with established melanoma if they were transiently depleted of host CD4^+^ T cells, given a tripartite ACT therapy and then treated with LPS. As shown in Fig. 8a, WT mice were first antibody depleted of CD4^+^ T cells prior to and during an ACT regimen and then treated with LPS one day after the cells were infused. Indeed, we found that the administration of LPS enhanced tumor destruction only in non-irradiated mice that were depleted of host CD4^+^ T cells (PFI + anti-CD4 + LPS > PFI + LPS or PFI + anti-CD4; Fig. 8b). Long-term cures (>70 days) were observed in these mice. Conversely, the depletion of CD4^+^ T cells alone or the activation of the innate immune system with LPS alone induces weak antitumor CD8^+^ T cell immune responses in non-irradiated animals with melanoma (Fig. 8b).

We were curious if depleting CD4^+^ T cells would impact the expansion of transferred CD8^+^ T cells. Although, the ratio of donor pmel-1 CD8^+^ T cells to host CD4^+^ T cells significantly increases when anti-CD4 antibody is given (Additional file 2), the number of CD8^+^ T cells did not change. It is possible that transiently removing host CD4^+^ T cells permits infused CD8^+^ T cells to better interface and be activated by APCs when mice are given LPS. Collectively, we speculate that depletion of host CD4^+^ T cells (which can function and cytokine sink or suppressors) plus LPS treatment bolster that antitumor properties (function and persistence) of infused pmel-1 CD8^+^ T cells in non-irradiated mice. Given the increased interest to determine and use novel neo-antigens to trigger effective T cell responses in patients with cancer [23], our data offers important insights into how and when to use TLR agonists to effectively augment T cell-based immunotherapies. Collectively, we identified the critical determinants for mediating successful ACT with TLR signaling and used this information to potentiate the antitumor activity of transferred CD8^+^ T cells with vaccination but without host preconditioning.

## Discussion

Lymphodepletion enhances adoptive immunotherapy via several reported mechanisms [40]. In addition to the removal of myeloid-derived suppressor cells, cytokine sinks and T_reg_ cells, translocation of gut microflora by TBI impacts the outcome of adoptive immunotherapy [11]. This intriguing finding that bacteria can promote tumor regression is reminiscent of Coley’s work with patients treated with repeated inoculations of erysipelas published in 1893 [41]. Yet, how to properly use bacteria or TLR agonists to most optimally enhance vaccines, checkpoint modulators and cellular therapy remains incompletely explored. Herein, we used TLR4 agonist LPS as a tool to address how and when to use TLR agonists to effectively improve cancer immunotherapy. We asked whether LPS could replace host lymphodepletion, vaccines or transferred CD8^+^ T cells in an aggressive model of melanoma.

In patients with advanced metastatic melanoma, a non-myeloablative regimen prior to infusion of tumor-infiltrating lymphocytes and bolus IL-2 resulted in an objective response rate of 50 % [42]. While the tolerated doses of lymphodepletion are well established, these systemic approaches are not devoid of toxicities. In mice, we report here that lymphodepletion to 5Gy TBI correlated with greater innate immune activation. Heightened innate activation was associated with greater impairment of the gastrointestinal tract, as evidenced by destruction of the colon and greater microbial LPS translocation [11]. However, we wished to find an effective and safe way to activate the innate immune system without compromising the GI tract. Thus, we administered LPS to non-irradiated mice receiving ACT. LPS could not augment ACT-mediated tumor regression in non-irradiated mice. However, low dose LPS could improve ACT treatment in mice given 5Gy TBI. Additional investigation revealed that LPS increased the expression of CD25 on pmel-1 CD8^+^ T cells infused into lymphodepleted mice. We are particularly intrigued by the fact that LPS robustly increased CD25 on donor pmel-1 CD8^+^ T cells in irradiated mice. Our new finding complements work from our colleague Mark Rubinstein. His lab recently published that the induction of CD25 on CD8^+^ T cells via IL-12 augments the engraftment potential and antitumor activity of adoptively transferred CD8^+^ T cells without host lymphodepletion [43]. Mechanistically, CD25 (i.e. the IL-2Rα) mediates IL-2 signaling and enhances immunotherapy. Based this new finding, we surmise that it is possible that LPS induced IL-12 by APCs in vivo. Thus, LPS-induced IL-12 possibly up-regulated CD25 on donor CD8^+^ T cells and in turn increasing the capacity of infused CD8^+^ T cells to consume homeostatic available IL-2 in the host. We are now conducting follow up studies in our lab to address this hypothesis.

Collectively, our data revealed that LPS alone is not enough to augment ACT treatment in non-irradiated animals consistent with findings that TBI provides multiple benefits to maximize ACT therapy, including TBI-mediated removal of immune cells by suppressive donor T cells. Moreover, beyond LPS, it is now well appreciated that a variety of microbial components induced by TBI/chemotherapy (or induced by other types of immunotherapies), can dramatically alter T cell-mediated tumor regression. Thus, it is likely that multiple TLRs, NODs, etc. on various innate immune cells are triggered, thereby enhancing T cell-based immunotherapies.

Importantly, we found that LPS doses toxic to non-irradiated mice were well tolerated in mice given low dose irradiation (5Gy TBI), possibly due to the fact that the number of APCs are reduced following irradiation [32] and therefore are unable to respond to LPS therapy. Furthermore, clinically available MPL and CpG-DNA was safe in 5Gy TBI mice and mediated CD8^+^ T cell-mediated destruction of tumors. Besides the difference in CpG sequence specificity, one important distinction between mice and humans is the expression pattern of TLR9 on immune cells. In humans, TLR9 is expressed only on pDC and B cells, whereas mice additionally express TLR9 on cells from the myeloid-lineage, resulting in a more dynamic response. All other effects of TLR9 ligands on human immune cells seem to be indirect and depend on factors produced by pDCs and B cells [44–46]. These differences may alter the translatability of CpG therapy (found in our work, Fig. 7b) between mouse and human. None the less, our data suggest that clinically available MPL, CpG, vaccines, or perhaps a recombinant human OX40 ligand or CD40 ligand could be used to safely activate the innate system [39, 47–49], thereby enhancing the infused tumor-reactive lymphocytes [50, 51]. Indeed, the engagement of the innate immune system as a trigger of the adaptive immune system represents a powerful approach to enhance adoptive immunotherapy. Moreover, it is likely that these therapies alter the contents of the microbiome, which many have recently published, have long-term and often positive consequences of mainstream checkpoint modulators [52, 53].

We found that administering LPS after a tripartite ACT therapy could improve tumor regression in irradiated (but not in non-irradiated) animals. However, additional investigation revealed that we could obviate the requirement for host preconditioning and drive curative responses in non-irradiated animals if we antibody ablated host CD4^+^ T cells transiently, along with ACT treatment followed by TLR agonist therapy. In retrospect, these findings are not surprising, as LPS has been shown to preferentially support the generation of regulatory T cells, known to suppress effector CD8^+^ T cells. Although some helper CD4^+^ T cells are known to bolster the cytotoxicity of CD8^+^ T cells, such as Th1 and Th17 cells, other non-specific CD4^+^ T cells impair the engraftment of infused CD8^+^ T cells by competing for cytokines induced by lymphopenia. Given that ablating CD4^+^ T cells enhances the antitumor therapy of pmel-1 CD8^+^ T cells, it would be interesting to execute these studies in Foxp3^DTR^ mice, eliminating FoxP3+ T regulatory cells. Ongoing studies in our laboratory are now focused on mechanistically understanding how this potent therapy is modulating the immune system to cure large established melanoma. We are also interested in understanding how and what type of host CD4^+^ T cells are reconstituting in these mice and if they are influencing the generation and memory biology of tumor-specific CD8^+^ T cells in vivo.

Finally, it is compelling to us that LPS could act either to enhance or hinder therapy depending on when it was delivered to the host. Though the LPS signal is meant to enhance the donor CD8^+^ T cells, particularly in the case of administering LPS before T cell infusion, it appears that this beneficial signal bolsters the generation of host immune cells over tumor specific T cells (Is this a phenomenon restricted to ACT therapy or does it impair vaccines, chemotherapy and/or checkpoint modulators as well?). Moreover, we also found that giving LPS during or prior to the tripartite regimen does not as robustly enhance the host DCs ability to drive the proliferation of the tumor-specific CD8^+^ T cells. In contrast, if LPS was given after the tripartite regimen, the host DCs effectively mediated the proliferation of pmel-1 CD8^+^ T cells in vitro. Based on these findings, our data argue that it is critical to consider when distinct therapies are given to a patient, particularly given that many investigators, including ourselves, believe that the path forward to improved treatment outcome in patients will be through combinatorial therapeutic approaches [54–57].

## Conclusion

Collectively, our results indicate that, though often overlooked in the design of cancer immunotherapy based clinical trials, that the temporal arrangement of multiple-treatment delivery is a paramount consideration in designing next generation T cell-based immunotherapies for cancer and infectious diseases. Specifically, our data identify how and when to administer TLR agonists to augment T cell-based immunotherapy in the absence or presence of host preconditioning.

## Methods

### Mice

To investigate the ability of TLR agonists to enhance ACT, we used the pmel-1 model to target B16F10 melanoma. All mice were bred and housed at NIH or MUSC facilities. Female pmel-1 TCR transgenic mice were crossed with C57BL/6-Ly5.1 Tg mice (C57BL/6-pmel-1-Ly5.1 mice; Jackson Laboratory). C57BL/6 and MyD88^-/-^ (Taconic) were used as recipients in ACT experiments. Experiments were conducted with the approval of the National Cancer Institue and Medical University of South Carolina Animal Use and Care Committee. The poorly immunogenic murine gp100^+^ B16F10 melanoma tumor was used.

### In vitro activation of pmel-1 CD8^+^ T cell generation

Pmel-1 splenocytes were cultured in the presence of 1 μM hgp100_25-33_ (KVPRNQDWL) with culture media containing 30 IU/ml of recombinant human IL-2 (rhIL-2; NCI preclinical repository), as described elsewhere [11]. Cells were transferred 7–10 days after the start of the culture.

### Regimen with adoptive transfer, vaccination, cytokines and TLR agonists

Recipient 6–10 week old mice were injected subcutaneously with 5e^5^ of the poorly immunogenic B16F10 tumor cells. Eight days later, mice received 0.5-1e^6^ in vitro activated pmel-1 CD8^+^ T cells. 5Gy TBI was given to mice the morning of ACT. CD4^+^ T cells were depleted by i.p. administration of 0.1 mg/mouse of anti-CD4 antibodies (BD Biosciences) -1, 1, 3, 5 and 7 days relative to cell transfer. Mice were vaccinated with 2e^7^ PFU of recombinant fowlpox virus expressing human gp100 (Therion Biologics). rhIL-2 was administered at 36 μg/dose 2X/day for five doses. Ultra-pure LPS (Invivogen; 0.1–50 μg, i.v.) was administered either 1 day pre, during or 1 day post PFI treatment. In some experiments, MPL (5 μg, i.v.) was given on day 1 following ACT. Alternatively, CpG (5’-TCCATGACGTTCCTGATGCT-3’) was given i.t. at 10 μg/day for 4 consecutive days. Experiments conducted in a randomized fashion and tumor measurements recorded over time by individuals unaware of treatment groups.

### Enumeration and phenotype of cells

At the indicated times, transferred pmel-1 cells were enumerated by multiplying the percent of Ly5.1/CD8^+^ T cells in the spleen by the absolute spleen count. Data were acquired on a BD LSRII (BD Biosciences) and analyzed using FlowJo software (Tree Star, Ashland, OR).

### *Ex vivo* proliferation assay

Untouched pmel-1 cells were isolated from splenocytes of an untreated animal, CFSE-labeled, and co-cultured at a 10:1 ratio with sorted CD11b^+^CD11c^hi^CD86^hi^ dendritic cells from tumor-bearing mice (day 6 post-ACT) given LPS at different time points. CFSE dilution was assayed on days 0, 2 and 4 post activation.

### Mucosal barrier score and detection of serum LPS

Colons were removed from mice and placed in 10 % formalin for 48 h, and then embedded in methylacrylate. 4–5 mm sections were taken along the papillary-optical axis. Sections were evaluated by a pathologist unaware of the identity of the groups using the scores as follows: normal architecture = 0, some signs of edema = 1, mild cell infiltration and reduction of crypts and goblets = 2, severe cell infiltration and profound reduction of crypts and goblets = 3, severe cell infiltration and visually undetectable crypt and goblets = 4. An LAL assay (QCL-1000; Cambrex) was used to analyze serum LPS on day 6.

### BrdU incorporation

Three days after treatment, mice were injected i.p. with 1 mg of BrdU (Sigma-Aldrich, St. Louis, MO). After two hours, the spleens from treatment groups were harvested, homogenized, and stained for pmel-1. Cells were permeabilized using Cytofix/Cytoperm (Pharmingen), treated with DNaseI (Sigma-Aldrich) for 1 h at 37 °C, then stained with FITC-conjugated anti-BrdU (BD Biosciences), and analyzed using FACS.

## Additional files

Additional file 1:
**One day after TBI, mice received an ACT treatment comprised of the adoptive transfer of 5e** ^**5**^ **cultured pmel-1 T cells, fowlpox hgp100 vaccination and hIL-2 or were left untreated.** Either on day during or one day after ACT, mice received 2 μg of LPS or were left untreated. Flow cytometry data shown are from splenocytes. (A) Percentage of CD25^high^FOXP3 + CD4^+^ T cells on day 5 following PFI. (B) Ratio of donor pmel-1 to host CD4^+^ T cells on day 5. ****P* < .001***P* < .01, **P* < .05, ANOVA. APCs were gated on either conventional DCs (C) or monocytes (D) on day 2 following PFI. (PDF 255 kb) Additional file 2:
**One day before ACT, mice were antibody depleted of host CD4** ^**+**^ **T cells (0.1 mg/treatment) and subsequently administered every other day for a total of 5 doses or left untreated.** The ACT treatment regimen was comprised of the adoptive transfer of 5e^5^ cultured pmel-1 T cells, fowlpox hgp100 vaccination and hIL-2 or were left untreated. One day after ACT, mice received 2 μg of LPS. The ratio of donor Vβ13 to host CD4 cells are shown from splenocytes on day 5 (mean ± SEM, 5 mice per group). *****P* < .0001, unpaired *t*-test. (PDF 70 kb)

## Abbreviations

ACT: Adoptive cell transfer
CpG: CpG oligodeoxynucleotides
HSC: Hematopoietic stem cell
LPS: Lipopolysaccharide
MPL: Monophosphoryl lipid A
PFI: P = infusion of 1e^6^ pmel-1 CD8^+^ T cells, F = vaccination with fowlpox encoding hgp100 and I = high dose IL-2
TBI: Total body irradiation
TLR: Toll-like receptor
WT: Wild-type
