# Supplementary figures and images for: Toll-like receptor agonist therapy can profoundly augment the antitumor activity of adoptively transferred CD8+ T cells without host preconditioning
Source: J Immunother Cancer. 2016 Feb 16;4:6. doi: 10.1186/s40425-016-0110-8 (PMC4754841; doi:10.1186/s40425-016-0110-8)

# Additional File 1

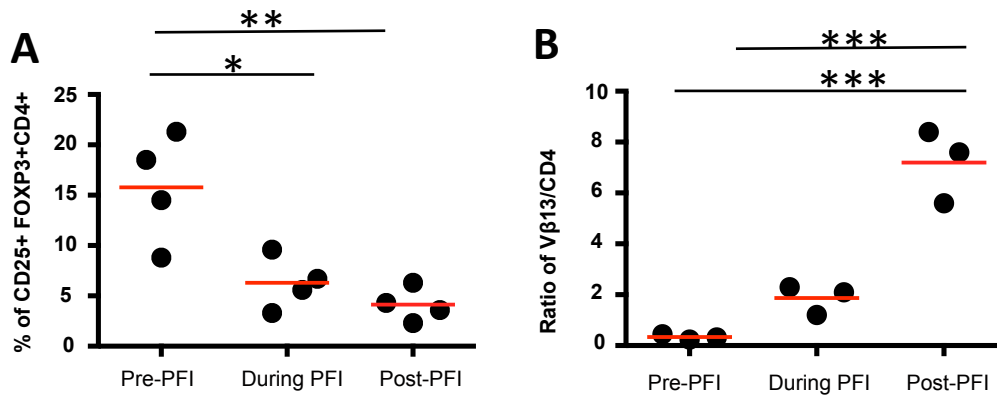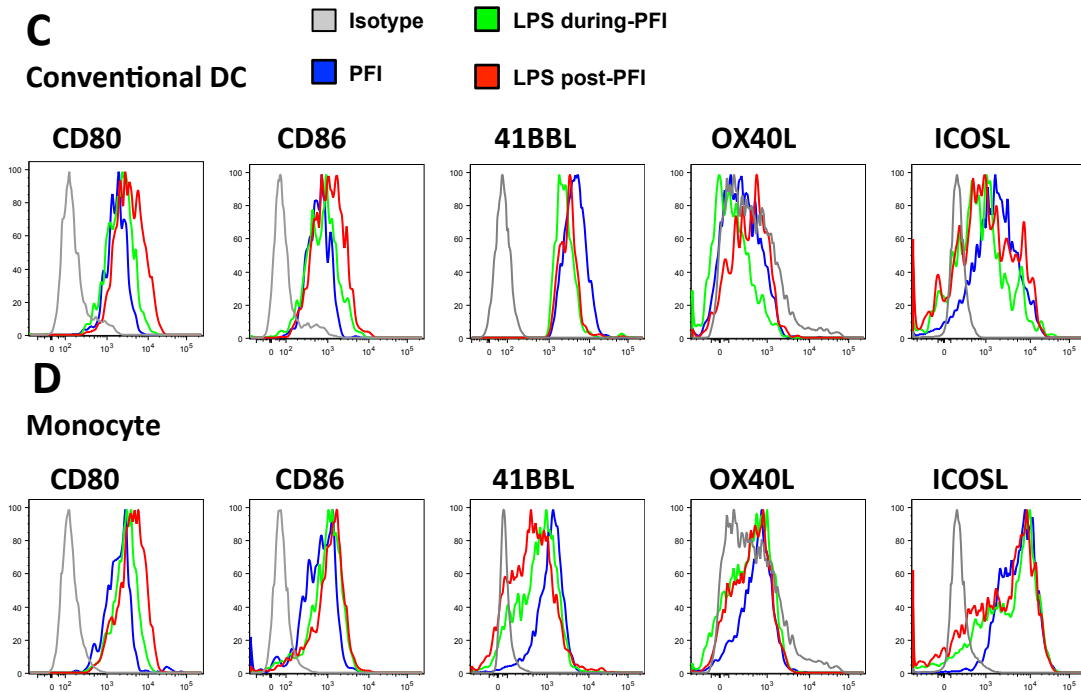

Supplement: Additional file 1: — One day after TBI, mice received an ACT treatment comprised of the adoptive transfer of 5e 5 cultured pmel-1 T cells, fowlpox hgp100 vaccination and hIL-2 or were left untreated. Either on day during or one day after ACT, mice received 2 μg of LPS or were left untreated. Flow cytometry data shown are from splenocytes. (A) Percentage of CD25highFOXP3 + CD4+ T cells on day 5 following PFI. (B) Ratio of donor pmel-1 to host CD4+ T cells on day 5. ***P < .001**P < .01, *P < .05, ANOVA. APCs were gated on either conventional DCs (C) or monocytes (D) on day 2 following PFI. (PDF 255 kb) [file 40425_2016_110_MOESM1_ESM.pdf]

## Additional File 2

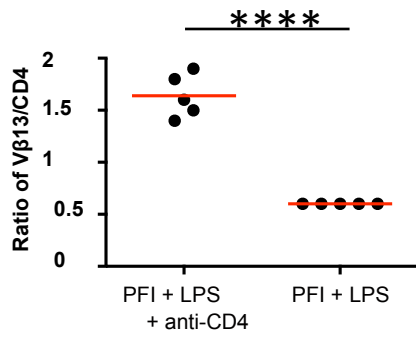

Supplement: Additional file 2: — One day before ACT, mice were antibody depleted of host CD4 + T cells (0.1 mg/treatment) and subsequently administered every other day for a total of 5 doses or left untreated. The ACT treatment regimen was comprised of the adoptive transfer of 5e5 cultured pmel-1 T cells, fowlpox hgp100 vaccination and hIL-2 or were left untreated. One day after ACT, mice received 2 μg of LPS. The ratio of donor Vβ13 to host CD4 cells are shown from splenocytes on day 5 (mean ± SEM, 5 mice per group). ****P < .0001, unpaired t-test. (PDF 70 kb) [file 40425_2016_110_MOESM2_ESM.pdf]
